# Defense Mechanisms and Borderline Personality Organization Among COVID-19 Believers and Non-believers During Complete Lock-Down

**DOI:** 10.3389/fpsyt.2021.700774

**Published:** 2021-08-24

**Authors:** Anna Zajenkowska, Iwona Nowakowska, Marta Bodecka-Zych, Joanna Rajchert, Izabela Kaźmierczak, Adrianna Jakubowska, Amy E. Pinkham

**Affiliations:** ^1^Institute of Psychology, The Maria Grzegorzewska University, Warsaw, Poland; ^2^Department of Psychiatry, University of Texas Southwestern Medical Center, Dallas, TX, United States; ^3^School of Behavioral and Brain Sciences, The University of Texas at Dallas, Richardson, TX, United States

**Keywords:** borderline personality organization, COVID-19 belief, denial, dissociation, splitting

## Abstract

The aim of the current study was to investigate whether a specific social perception of the pandemic—believing or not in COVID-19—predicts borderline personality organizations and whether this relationship is mediated by more primitive maladaptive mechanisms—splitting, denial, and dissociation. The online study included 720 organization aged 25–45. Participants were diverse in terms of place of residence, being in a relationship, and education level. Approximately 30% of the general population reported not believing in the COVID-19 pandemic. Non-believers scored slightly higher on borderline symptoms and used more maladaptive defense mechanisms than believers. Individuals who deny COVID-19 are more likely to show characteristics of borderline personality organization. Splitting is an important mechanism in this relationship.

## Introduction

Perceptions and attitudes toward negative and powerful life events can be related to the type of defense mechanisms an individual employs and the level of functioning of the ego (1). To many people a sudden shift in their everyday lives due to the recent COVID-19 pandemic and lock-down could have been a traumatizing event and may have triggered the use of maladaptive defense mechanisms (2, 3). That could be especially true among individuals with weaker ego, who use more maladaptive defense mechanisms (4). It is plausible that the way an individual perceives reality under stressful events could enhance pathological functioning if he/she uses maladaptive defense mechanisms. A highly stressful event like the pandemic can increase support for the ideology, convictions and possibly defense mechanisms that were already embraced before the stressor appeared (5, 6).

Defense mechanisms are related to the way people process everyday events. The way people interpret certain situations creates behavioral patterns, and the greater the cross-situational consistency, the more that constitutes a certain personality characteristic (7, 8). If a situation is strong [meaning salient, guiding behaviors so that people construe it in similar ways, (9, 10), the way people perceive it predicts certain behaviors to a greater extent than just personality characteristics (11, 12). Hence, in line with socially constructed perspective, questions in psychology should be answered not only in regard to psychological inner states (e.g., experienced feelings or dispositional factors), but also with consideration of an individual in interaction with a situational context (13, 14). In line with this perspective, psychopathology and mental disorder can be constructed by specific situational perceptions of an individual. Thus, perhaps, if an individual experiences chronic stressors (such as the prolonged pandemic situation) and continually chooses maladaptive defense mechanisms, it can lead to the development of psychopathological symptoms. Furthermore, if a person who is already displaying mental health problems finds themselves in such a situation, it can reinforce the choice of such defenses.

The aforementioned assumptions could shed different light on the borderline personality organization during pandemic. Those with borderline personality organization process reality using mainly splitting, primitive denial, or projective identification (4, 15), also individuals diagnosed with borderline personality disorder (BPD) report using a maladaptive and image-distorting defense style more often as compared to non-BPD individuals (16). The function of maladaptive mechanisms is in most cases an adaptation to stressful, traumatic or unbearable events (17). Among them, a few have a distinct function to either block the events from awareness, like denial or dissociation, which is an emotional detachment from reality, or to deal with the ambiguity or uncertainty of events but polarizing views—that is mainly splitting (4, 18). For these reasons, we tested whether believing or not in COVID is associated with borderline personality symptoms and whether that is related to using the abovementioned more primitive maladaptive defense mechanisms.

In general, we propose that particular perceptions of an event may be linked to psychopathology. In the current study we were curious whether a specific social perception of the pandemic, namely rejecting the idea of the COVID pandemic during a complete lock-down, is related to deeper psychological dysfunction in the form of utilizing those maladaptive defense mechanisms that are commonly seen in BP symptomatology. These mechanisms all aim to block immediate reality and include dissociation, denial and splitting, with the latter involving also a fundamental lack of integration and black-and-white thinking (19).

## Method

### Participants and Procedure

A total of 720 volunteers aged 25–45 (*M* = 34.37, SD = 5.71) participated in this study (71,1% female, 27,9% male; 1 person refused to state their gender). The sample was not fully representative for the Polish population given the restricted age range and gender distribution, however, it was diverse in terms of place of residence, being in a relationship, and education level (Table 1). Sixty four (8.9%) respondents reported being currently in psychotherapy, and 49 (6.8%) reported taking medication prescribed by a psychiatrist. All participants were recruited by a Polish online research pool Ariadna. Convenience sampling was applied, given that only people who chose to register for the research pool were able to take part (an invitation was sent to them by the pool mailing system).

**Table 1:** Demographic and psychometric characteristics of believers and non-believers; for numeric variables means and standard deviations in parenthesis with *t*-tests (or Welch tests) and Cohen's *d* were presented and for dichotomous variables (sex, compliance, borderline > 10) number and percentage in parenthesis with *X*^2^ test and Cramer's V was presented.

**Variable**	**Believers *N* = 504 *M* (SD) or N (%)**	**Non-believers *N* = 216 *M* (SD) or *N* (%)**	***t* or *X^**2**^***	***p***	**Cohen's *d* or Cramer's V**
Age	34.68 (5.84)	33.66 (5.33)	2.27	0.023	0.18
Sex (women)	355 (70.60)	163 (75.50)	1.79	0.18	0.05
Compliance (yes)	491 (97.40)	164 (75.90)	85.60	<0.001	0.34
Borderline	5.06 (4.30)	5.98 (4.93)	−2.35	0.019	−0.20
Borderline > 10	54 (10.70)	35 (16.20)	4.20	0.040	0.08
Anxiety	14.12 (3.93)	13.95 (3.80)	0.53	0.59	0.04
Depression	13.36 (4.10)	13.31 (4.17)	0.14	0.88	0.01
Denial	6.89 (3.19)	7.62 (3.27)	−2.79	0.005	−0.22
Dissociation	7.31 (3.06)	8.08 (3.39)	−2.98	0.003	−0.24
Splitting	8.96 (3.62)	9.92 (3.76)	−3.22	0.002	−0.26

The study was conducted in October-November 2020 in Poland, during a period of the “second wave” of the COVID-19 pandemic. At that time, the number of cases had been increasing from moderate to high (20). No vaccines were then available in Poland. The study was a part of a larger research project, but the analyses reported here are completely novel. It was conducted online and consisted of a series of self-report questionnaires.

Statistical power was calculated with G^*^Power 3.1 analyses (21). According to this, our sample size allowed for detection of an effect of partial *R*^2^ increase of.05, α = 0.05 with a power of 0.99. All analyses were conducted using IBM SPSS 26 for Windows with Andrew F. Hayes 3.4.1 macro for SPSS (22).

### Measures

To measure borderline symptoms we used the Polish adaptation^1^ of the Borderline Personality Inventory [BPI; (23)]. Its short version consists of 22 true-false items which refer to the diagnostic criteria of borderline personality disorder. The BPI identifies patients with borderline personality organization in high agreement with the clinical criteria as well as with the Gunderson's criteria for BPD. It is recommended as an instrument to assess the borderline personality organization, BPD, and borderline features in disorders from Axis I and II (24). However, it ought to be noted that the measure is self-report based and provides insight into symptomatology, but it cannot be considered equivalent to observer-based clinical diagnosis. The borderline personality indicator was created by summing the “true” responses, each such response is 1 point. The cutoff point for borderline personality is 10 points. The measure had a satisfactory reliability in our study (Cronbach's α = 0.86).

To measure defense mechanisms, the Polish version (1) of Defense Style Questionnaire-40 [DSQ-40; (25)] was used. It assesses 20 defense mechanisms andconsists of 40 items (two items per each mechanism). Participants responded by indicating how much they agree with each item using a 9-point Likert scale (1= *completely disagree*, 9 = *completely agree)*. In the current study, three mechanisms were of interest: splitting (Cronbach's α = 0.48), denial (Cronbach's α = 0.50) and dissociation (Cronbach's α = 0.51).

To measure believing in COVID-19 we used a question asking “Do you believe in the global coronavirus pandemic of SARS-CoV-2?.” The participants marked their answers on a yes-no scale.

## Results

Results showed that there were more believers (*N* = 504, 70%) than non-believers (*N* = 216), χ^2^ (1) = 115.20, *p* < 0.001. Comparison between believers and non-believers indicated that number of men and women was similar. However, believers were lower on borderline symptoms scale, less often exceeded the cutoff for the borderline organization diagnosis and used less Denial, Dissociation and Splitting mechanisms than non-believers. Table 1 presents means and frequencies of study variables in believers and non-believers with comparison statistics.

Further analysis showed that greater Borderline symptoms were related to Splitting, *r* = 0.30, *p* < 0.001, Denial, *r* = 0.12, *p* = 0.002 and Dissociation, *r* = 0.13, *p* = 0.001, and Dissociation and Denial were closely related to each other, *r* = 0.62, *p* < 0.001 and less strongly to Splitting (Dissociation, *r* = 0.26, *p* < 0.001, Denial, 0.34, *p* < 0.001). Next, three separate mediation models were tested using PROCESS version 3.4.1. macro (22), where non-believing (0—believing; 1—non-believing) was the predictor of borderline symptoms and one of the three defense mechanisms was the mediator. In Table 2, coefficients for each model are presented.

**Table 2:** Coefficient in models testing relationship between believing vs. non-believing in COVID and borderline symptoms mediated through splitting, denial, and dissociation.

	**Coeff**	**SE**	***t***	***p***	**95% CI**	**Stand. Coeff**
**Mediation through splitting**						
Non-believing and splitting relation	0.96	0.29	3.22	0.001	0.37, 1.54	0.26
Splitting and borderline relation controlling for non-believing	0.35	0.04	8.17	<0.001	0.27, 0.44	0.29
Non-believing and borderline relation controlling for splitting	0.56	0.35	1.60	0.108	−0.12, 1.26	0.12
**Mediation through denial**						
Non-believing and denial relation	0.73	0.26	2.79	0.005	0.21, 1.24	0.22
Denial and borderline relation controlling for non-believing	0.15	0.05	2.94	0.003	0.05, 0.25	0.10
Non-believing and borderline relations controlling for denial	0.80	0.36	2.18	0.029	0.08, 1.51	0.17
**Mediation through dissociation**						
Non-believing and dissociation relation	0.76	0.25	2.98	0.003	0.26, 1.27	0.24
Dissociation and borderline relations controlling for non-believing	0.17	0.05	3.22	0.001	0.06, 0.27	0.12
Non-believing and borderline relation controlling for dissociation	0.78	0.36	2.13	0.033	0.06, 1.50	0.17

The total effect of Non-believing on Borderline symptoms was significant, *B* = 0.91, SE = 0.36, *t* = 2.49, *p* = 0.013, 95% CI [0.19, 1.63], β = 0.20. Results showed that all three mechanisms partially mediated this effect, Splitting, *B* = 0.34, SE = 0.11, 95% CI [0.13, 0.56], β = 0.07, Dissociation, *B* = 0.13, SE = 0.06, 95% CI [0.02, 0.27], β = 0.03, Denial, *B* = 0.11, SE = 0.05, 95% CI [0.01, 0.23], β = 0.02. The direct effect became non-significant when Splitting was included, but was still significant when Denial and Dissociation effects were accounted for.

## Discussion

The current study shows that ~30% of the sample reported not believing in the COVID pandemic. Non-believers scored slightly higher on borderline symptoms (*d* = 0.20) and used more maladaptive defense mechanisms than believers. The association between borderline personality symptoms and COVID denial became non-significant after controlling for both their associations with splitting (but not with denial and dissociation), highlighting the possibility that splitting is responsible for the link between the two. These findings confirm that splitting is a psychological defense that is considered a central marker of borderline personality disorder symptoms (26).

The COVID-19 virus is life-threatening, and the pandemic constitutes both an intensive and chronic stressor. In such demanding situations the ego's defenses may weaken or even collapse, leading to decompensation (27–29). Such a failure could result in extreme anxiety and the ultimate defense of the ego from annihilation, which could be dissociation. It is characterized by feeling cut off from oneself, seeing oneself from outside one's body, or feelings of unreality (4). To prevent this state and defend against what one experiences as an overwhelming event or trauma, the ego might use splitting (30). This is an exact defense mechanism against extreme anxiety that is related to dissociation and death. Patients compartmentalize memory: one part of the ego may stay in touch with the non-disturbing reality while the other one may lose this contact and reject all aspects that are viewed as too distressing. The individual might even construct an alternative, more desirable reality (30).

Maintaining this kind of maladaptive coping across time may lead to an inability to create an abiding sense of self and/or significant impairments in the ego (31). The ego is poorly developed or with unstable self-image as the ego is built “between two worlds.” In this sense, perception of social events may “create” the psychopathology, and further weaken the unstable ego leading to deepening the symptoms of BPD. That notion has to be explored however in longitudinal studies. In a manner of speaking, if the environment is traumatic and full of “strong” events, people may, by using more primitive defenses, “build up” personality disorder. Investigating such idea might have great implications for clinical work—changing the environment and replacing defense mechanisms, which become non-adaptive in a new situation, may lead to improvement in PDs. The described above association between denying or minimizing the event and borderline personality organization may go beyond the COVID-19 related phenomena and be universal. As Minikin (32) shows, intolerance feeds regressive defenses such as splitting, which relates to alienation. The latter is viewed as the root cause of all mental and social distress.

Some limitations require consideration. While the current investigation clearly establishes a relationship between denial of COVID and increased symptoms of borderline personality symptomatology, these results are correlational and cross-sectional and cannot address causality. The borderline personality symptomatology was assessed only with a self-report questionnaire, which while reliable does not allow clinical diagnoses. The participants were recruited by a research pool, enabling us to gather a diverse sample in terms of place of residence, age or professional background, however, the study cannot be considered representative for the Polish population. Additionally, our question regarding belief in the COVID-19 pandemic was straightforward but was not able to assess any nuances in regard to this belief. Future studies would likely benefit from a more thorough assessment of COVID-19 beliefs. Notwithstanding these limitations, the current findings indicate that those individuals who deny COVID-19 are more likely to show characteristics of borderline personality symptomatology and that splitting is an important mechanism in this relationship.

## Data Availability Statement

The datasets presented in this study are published open access and can be found online. They can be downloaded from: http://apsycholab.pl/downloads/.

## Ethics Statement

Ethical review and approval was not required for the study on human participants in accordance with the local legislation and institutional requirements. The patients/participants provided their written informed consent to participate in this study.

## Author Contributions

AZ: conceptualization, methodology, investigation, resources, writing—original draft, writing—review and editing, supervision, and project administration. IN: investigation, resources, writing—original draft, writing—review and editing, and project administration. MB-Z: investigation, writing—original draft, and writing—review and editing. JR: methodology, formal analysis, investigation, data curation, writing—original draft, and visualization. IK: investigation, resources, and writing—original draft. AJ and AP: writing—original draft and writing—review and editing. All authors contributed to the article and approved the submitted version.

## Conflict of Interest

The authors declare that the research was conducted in the absence of any commercial or financial relationships that could be construed as a potential conflict of interest.

## Publisher's Note

All claims expressed in this article are solely those of the authors and do not necessarily represent those of their affiliated organizations, or those of the publisher, the editors and the reviewers. Any product that may be evaluated in this article, or claim that may be made by its manufacturer, is not guaranteed or endorsed by the publisher.

## Footnotes

^1^Cierpialkowska L. Adaptacja Kwestionariusza Osobowosci Borderline F. Leichsenringa [Polish adaptation of F. Leichsenring Personality Inventory] (2001).
